# Oxidative phosphorylation activation is an important characteristic of DOX resistance in hepatocellular carcinoma cells

**DOI:** 10.1186/s12964-018-0217-2

**Published:** 2018-02-05

**Authors:** Li Wu, Jiayu Zhao, Kexin Cao, Xiao Liu, Hao Cai, Jiaqi Wang, Weidong Li, Zhipeng Chen

**Affiliations:** 1 Department of Pharmacology, School of Pharmacy, Nanjing University of Chinese Medicine, Nanjing, Jiangsu People’s Republic of China; 2 Jiangsu Key Laboratory for Pharmacology and Safety Evaluation of Chinese Materia Medica, Nanjing University of Chinese Medicine, Nanjing, Jiangsu People’s Republic of China; 3 Engineering Center of State Ministry of Education for Standardization of Chinese Medicine Processing, School of Pharmacy, Nanjing University of Chinese Medicine, Nanjing, Jiangsu People’s Republic of China; 4 Affiliated Hospital of Integrated Traditional Chinese and Western Medicine in Jiangsu Province, Nanjing University of Chinese Medicine, Nanjing, Jiangsu People’s Republic of China

**Keywords:** Drug resistance, Hepatocellular carcinoma, Mechanism, α-ketoglutaric acid, Energy metabolism

## Abstract

**Background:**

Despite the implications for tumor growth and cancer drug resistance, the mechanisms underlying differences in energy metabolism among cells remain unclear.

**Methods:**

To analyze differences between cell types, cell viability, ATP and α-ketoglutaric acid levels, the oxygen consumption rate and extracellular acidification rate, and the expression of key enzymes involved in α-KG metabolism and transfer were examined. Additionally, UPLC-MS/MS was used to determine the doxorubicin (DOX) content in SMMC-7721 and SMMC-7721/DOX cells.

**Results:**

We found that energy metabolism in SMMC-7721 cells is mainly dependent on the glycolysis pathway, whereas SMMC-7721/DOX cells depend more heavily on the oxidative phosphorylation pathway. Cell viability and intracellular ATP levels in SMMC-7721/DOX cells were significantly reduced by rotenone and oligomycin, inhibitors of oxidative phosphorylation. However, SMMC-7721 cell properties were more strongly influenced by an inhibitor of glycolysis, 2-deoxy-d-glucose. Furthermore, the suppressive effect of α-KG on ATP synthase plays an important role in the low levels of oxidative phosphorylation in SMMC-7721 cells; this effect could be strengthened by the metabolic poison methotrexate and reversed by l-(−)-malic acid, an accelerator of the malate-aspartate cycle.

**Conclusions:**

The inhibitory effect of α-KG on ATP synthase was uncoupled with the tricarboxylic acid cycle and oxidative phosphorylation in SMMC-7721 cells; accordingly, energy metabolism was mainly determined by glycolysis. In drug-resistant cells, a remarkable reduction in the inhibitory effects of α-KG on ATP synthase resulted in better coordination among the TCA cycle, oxidative phosphorylation, and glycolysis, providing novel potential strategies for clinical treatment of liver cancer resistance.

## Background

The reprogramming of energy metabolism, including aerobic glycolysis and the inhibition of mitochondrial energy metabolism, promotes tumor growth [1–8]. Additionally, it is closely related to multidrug resistance (MDR) in chemotherapy [9, 10]. The effect of mitochondrial energy metabolism on MDR is complex and research progress in this area is slow [11].

The inhibition of mitochondrial energy metabolism in hepatocellular carcinoma cells shows significant plasticity. The process is only partially and reversibly inhibited in normal conditions and is reactivated under stress conditions, e.g., under a lack of oxygen and chemical therapy, and is involved in MDR. Further studies of its proximal mechanisms can provide new targets for reversing MDR [12–17]. According to previous studies, tricarboxylic acid cycle (TCA) enzyme activity in AS-30D hepatoma cells is higher than typical activity levels in non-cancer cells and there is no change in mitochondrial electron transport chain function, indicating that the TCA cycle is not significantly suppressed in these cells [14]. However, it is not clear how glycolysis affects the TCA cycle and oxidative phosphorylation (OXPHOS) or how adenosine triphosphate (ATP) production is reduced in cases where the TCA cycle is constant or even enhanced. Therefore, it is necessary to study characteristic changes in glycolysis and mitochondrial energy metabolism to determine the mechanisms underlying the reactivation of mitochondrial energy metabolism.

Oxaloacetic acid metabolized by lactic acid in hepatocyte mitochondria can enter the TCA cycle, and can be metabolized into aspartic acid by ammonification to enter the gluconeogenic pathway to generate glucose. When the gluconeogenic pathway is blocked, oxaloacetic acid enters the TCA cycle to maintain a normal mitochondrial membrane potential difference and morphology [18]. Studies have shown that there are no significant differences in the function of the complexes (I, II, III, and IV) that maintain mitochondrial membrane potential [19, 20]. However, complex V (ATP synthase) often exhibits abnormal expression or function, and this is considered an important factor in mitochondrial energy metabolism dysfunction [13]. However, the specific mechanisms, particularly the mechanism underlying the reactivation of mitochondrial energy metabolism after chemotherapy, have not been clarified.

A complete loss of mitochondrial function is bad for normal cells, but partial inhibition of the electron transport chain can prolong the life of cells, and this is commonly observed in low-grade and mammalian cells [21]. α-Ketoglutaric acid (α-KG), an intermediate in the TCA cycle, can reduce ATP production by inhibiting the activity of ATP synthase, without affecting electron flow through the electron transport chain (mediated by complexes I, II, III, and IV); it has an antiapoptotic role by increasing mitochondrial autophagy [22–24].

Recent research has shown that α-KG is not just a regulator of many energy metabolism enzymes, but is also a precursor of a variety of amino acids involved in cell metabolism. However, it is not clear whether the inhibitory effects of α-KG on ATP synthase also occur in hepatoma carcinoma cells. In this study, SMMC-7721 and doxorubicin (DOX)-resistant SMMC-7721/DOX cells were used to investigate resistance mechanisms from the perspective of energy metabolism.

## Methods

### Establishment of a drug-resistant cell model and cell culture

Continuous induction by applying low-dose DOX with increasing concentrations to SMMC-7721 cells was used to generate drug-resistant cells. The cells were grown in medium with 0.43 μM DOX until resistance was acquired, and resistant cells are referred to as SMMC-7721/DOX. They were cultured under standard cell culture conditions with 10% fetal bovine serum with 1% penicillin-streptomycin at 37 °C in a humidified incubator with 5% CO_2_.

### In vitro cytotoxicity test

Cells (2 × 10^4^) were seeded in 96-well plates, and the growth curve was recorded by the IncuCyte real-time video imaging system (Essen Instruments, Ann Arbor, MI, USA). In the cytotoxicity test, cells were seeded in 96-well plates, allowed to attach overnight, and treated with DOX (0.1–40 μM) for 24 h. Cell viability was measured by the MTT method and the IC50 of DOX was calculated using Graphpad prism. The resistance index (RI) was calculated as follows: RI = IC50 (SMMC-7721/DOX)/IC50 (SMMC-7721).

### Determination of ATP and protein levels

SMMC-7721 and SMMC-7721/DOX cells were administrated DOX and other drugs for 24 h. Endogenous expression of ATP in lysates of treated cells was detected using an Enhanced ATP Assay Kit following the manufacturer’s instructions (093016161116; Shanghai Biyuntian Biological Co., Ltd., Shanghai, China).

### Mitochondrial stress test and glycolysis stress test

Cells (2 × 10^4^) were seeded in 96-well plates, cultured for 24 h to achieve 80% coverage, and treated with drugs at various time points. The oxygen consumption rate (OCR) in the mitochondrial stress test and extracellular acidification rate (ECAR) in the glycolysis stress test were determined using the Seahorse-XF 96 (Seahorse Bioscience, North Billerica, MA, USA), and data were analyzed using Seahorse Wave.

### Determination of α-KG

The α-KG content was determined using commercially available kits according to the manufacturer’s protocols (#3C09K06770; Sigma, St. Louis, MO, USA). Briefly, 2 × 10^6^ cells were homogenized in 100 μL of ice-cold α-KG buffer. Samples were centrifuged at 13,000×*g* for 10 min to remove insoluble material, adjusted to a final volume of 50 μL with α-KG assay buffer, and deproteinized with 10-kDa MWCO (Millipore, Billerica, MA, USA) spin filter before addition to the reaction to prevent interference from enzymes in the samples. Reactions consisted of 44 μL of the sample or standard, 2 μL of α-KG converting enzyme, 2 μL of α-KG development enzyme mix, and 2 μL of fluorescent peroxidase substrate; they were incubated at 37 °C for 30 min. The absorbance of each reaction system was measured at 570 nm (A_570_) on a microplate reader.

### Immunofluorescence analyses

Cells (5 × 10^3^) were cultured in 6-well Merck Millicell EZ slides (Merck Millipore, Darmstadt, Germany), allowed to attach overnight, and treated with DOX for 24 h. Afterwards, the slides were washed twice in PBS, fixed in 4% formaldehyde in PBS (10 min, room temperature) and incubated with 0.2% Triton X-100 in PBS (10 min, room temperature). The fixed cells were incubated overnight at 4 °C with specific antibodies. Protein expression was detected using mouse monoclonal Ab against P-gp. The primary antibodies were detected after 1 h of incubation with anti-rabbit HRP-conjugated antibodies at a dilution 1:2000 in antibody diluent. Finally, the slides were washed 3 times in PBS and Pro Long Gold Mounting Medium with DNA intercalating dye 4,6-diamidino-2-phenylindole (DAPI) was added to visualize the cell nucleus. The analysis was conducted under fluorescence microscope.

### Western blot analysis

Cell extracts were acquired from treated SMMC-7721 and SMMC-7721/DOX with RIPA buffer plus proteinase inhibitors. Proteins were resolved by electrophoresis on SDS-polyacrylamide gels and transferred to a polyvinylidene fluoride membrane (Millipore). Proteins of interest were detected using specific primary antibodies, followed by specific secondary antibodies. The expression of proteins of interest was analyzed using ImageJ (NIH, Bethesda, MD, USA). Changes in the density of bands are expressed as fold changes compared to the control in the blot after normalization to β-actin.

### Determination of intracellular DOX by UPLC-MS/MS

RIPA buffer (100 μL) was added to cells after treatment for 24 h. The protein in cell lysates was precipitated by methanol, and the supernatant after high-speed centrifugation (12,000 *g*, 10 min, 4 °C) was dried with nitrogen and re-dissolved in methanol. The supernatant after high-speed centrifugation was directly injected into the UPLC-MS/MS system. This system was a Shimadzu UPLC system equipped with a LC-30 AD binary pump, an on-line degasser (DGU-20A5R), an auto-sampler (Model SIL-30SD), a column temperature controller compartment (CTO-30A), and a 5500 Triple Quad Tandem Mass Spectrometer (AB Sciex, Concord, Ontario, Canada) with an electrospray ionization (ESI) source. Analytes were separated using an Extend C18 column (2.1 mm × 100 mm, 1.8 μm; Agilent, Santa Clara, CA, USA). The mobile phase was composed of a mixture of 1% formic acid water (A) and acetonitrile (B) and a gradient elution program was used (0–2.5 min, 15% B to 40% B, 2.5–4.0 min, 40% B, 4.0–4.1 min, 40% B to 95% B, 4.1–5.0 min, 95% B, 5.0–5.1 min, 95% B to 15% B, 5.1–6.6 min, 15% B). The flow rate was set at 0.3 mL/min, the column temperature was 40 °C, and the injection volume was 2 μL. The ESI source was operated in positive ionization mode. The mass spectrometer was operated in multiple reactions monitoring (MRM) mode. The MS parameters of DOX are presented in Table 1. The optimized parameters were as follows: ion source temperature, 550 °C; curtain gas, 35 psi; ion source gas 1, 55 psi; ion source gas 2, 55 psi; ion spray voltage, 5500 V.

**Table 1:** Optimized multiple reaction monitoring (MRM) parameters for DOX

Compounds	Q1	Q3	CE/V	DP/V	EP/V
DOX	544.3	397.1	15.03	57.05	10.00

### Statistical analysis

Data are presented as means ± standard deviation (SD). One-way analysis of variance (ANOVA) and *t*-tests were used for comparisons between groups. All statistical analyses were implemented in SPSS 15.0 with a significance threshold of *p <* 0.05.

## Results

### Low-dose DOX-induced drug resistance in hepatoma SMMC-7721 cells

**Fig. 1:**
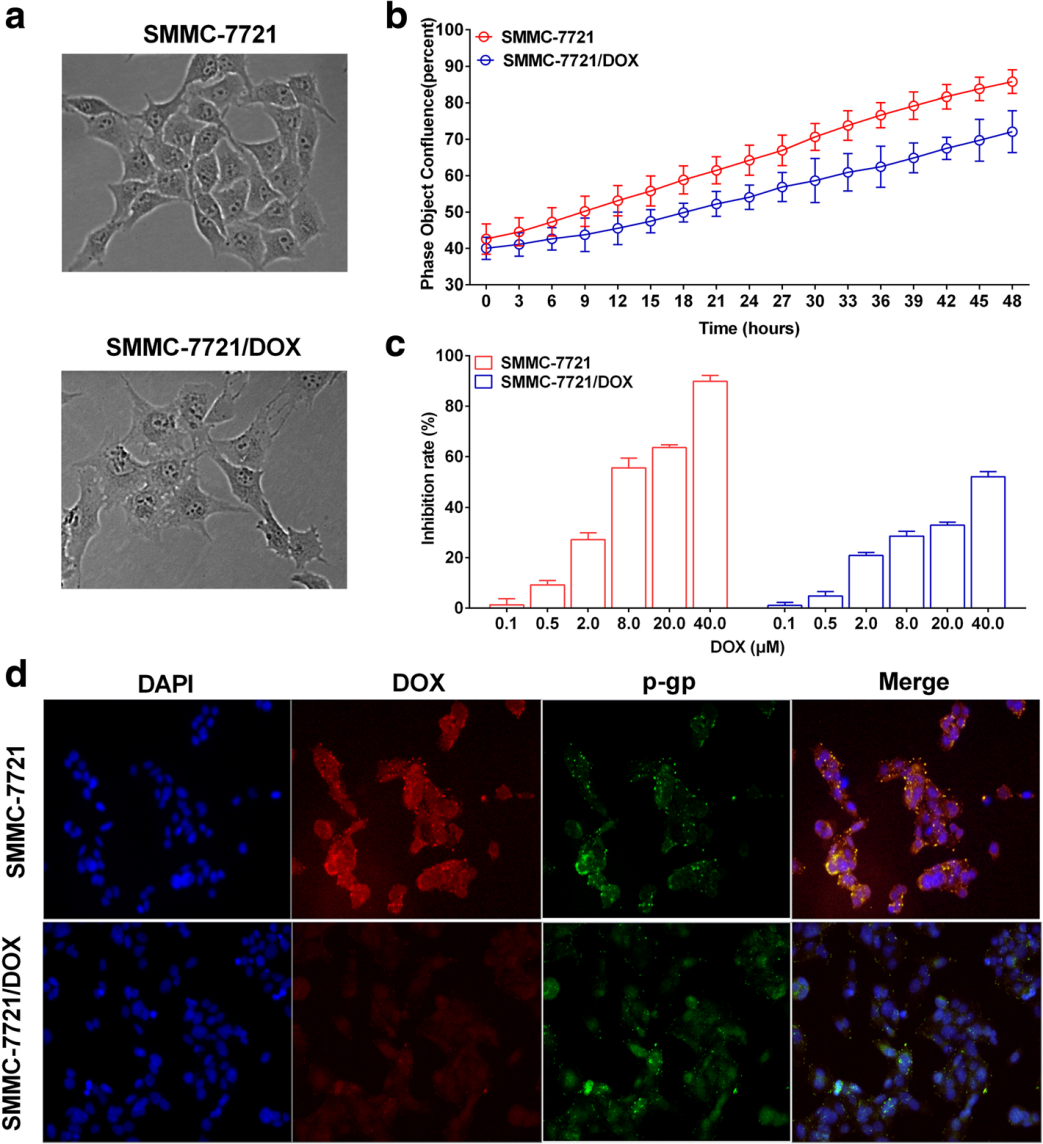
Low-dose DOX-induced drug resistance in hepatoma SMMC-7721 cells. **a** SMMC-7721 and SMMC-7721/DOX cell configuration. **b** Growth curves of SMMC-7721 and SMMC-7721/DOX cells **c** Change in cell viability induced by different concentrations of DOX. **d** Immunofluorescence staining for P-gp with polyclonal mouse anti-P-gp minigene (green). Nuclear DNA was stained with DAPI (blue). DOX showed red fluorescence. Merge, maximum projection

Treatment of SMMC-7721 cells with low-dose DOX at increasing concentrations was successfully used to establish drug-resistant cells. Compared with the sensitive cells, SMMC-7721/DOX cells showed increased slightly volume, relatively irregular shape, unequal size, unclear border, and more intracellular granules (Fig. 1a). The 48-h growth curve showed slightly delayed proliferation of drug-resistant cells (Fig. 1b). In the cytotoxicity test, SMMC-7721 and SMMC-7721/DOX cells exhibited significant differences in viability to DOX at doses ranging from 0.1 to 40 μM (Fig. 1c). The IC50 values of DOX in SMMC-7721 and SMMC-7721/DOX cells were 23.27 μM and 185.00 μM, respectively, and the RI for SMMC-7721/DOX was 7.95.

DOX is a type of anthracycline antitumor drug and exhibits red fluorescence. The fluorescence intensity and relative dose of DOX in cells showed a linear relationship; thus, we analyzed the red fluorescence in SMMC-7721 and SMMC-7721/DOX cells by fluorescence microscopy. After 24-h incubation, the amount of DOX in SMMC-7721/DOX cells was more than that in SMMC-7721 cells. However, the expression of P-gp did not show any significant difference between sensitive and resistant cells (Fig. 1d). These results suggest that the expression of P-gp was not the main mechanism of the decreasing in DOX content in SMMC-7721/DOX cells.

### Difference in energy metabolism mode between SMMC-7721 and SMMC-7721/DOX cells

**Fig. 2:**
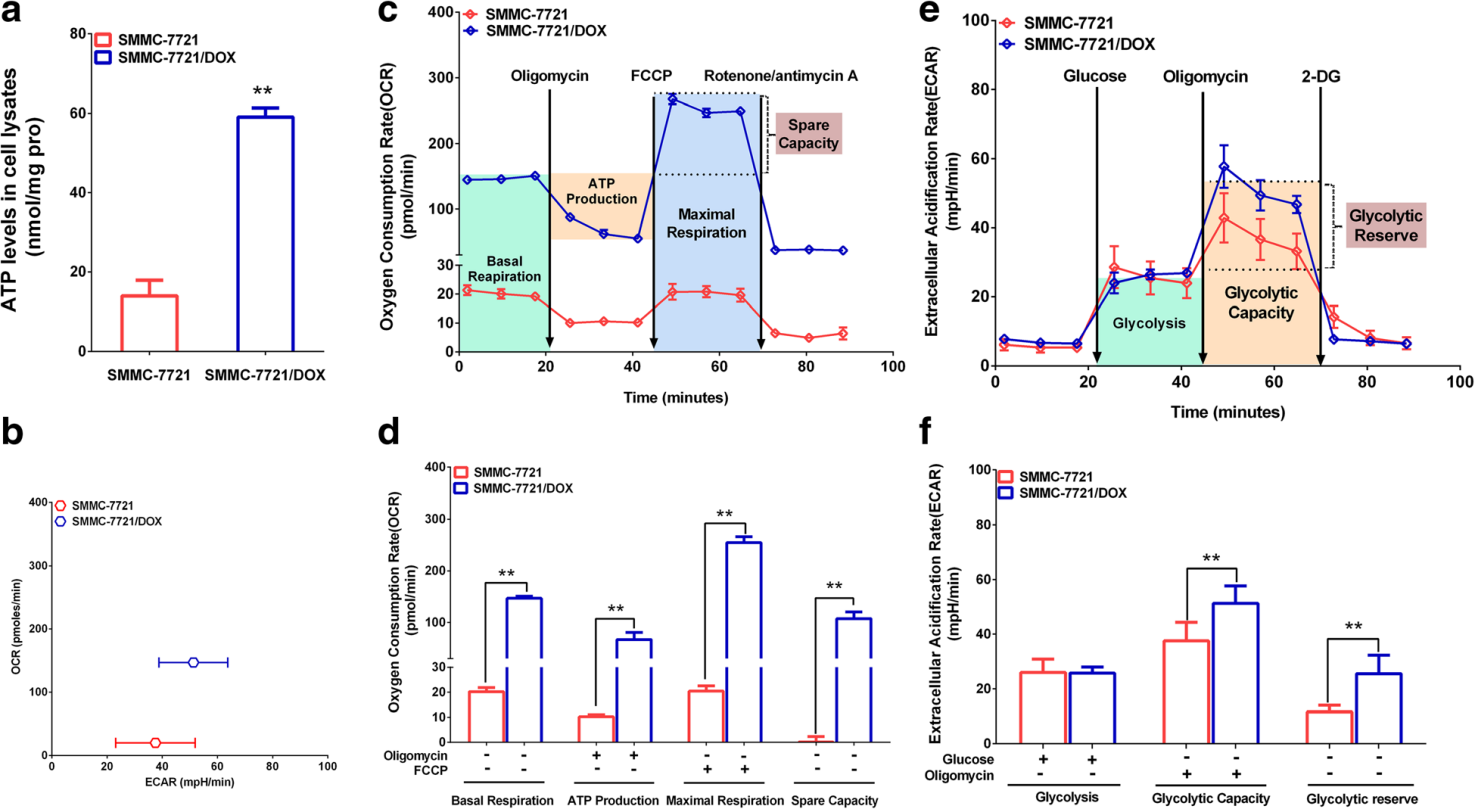
Differences in ATP levels and energy metabolism mode between SMMC-7721 and SMMC-7721DOX cells. **a** Intracellular ATP levels in SMMC-7721 and SMMC-7721/DOX cells. **b** Cell respiration (OCR) against glycolysis (ECAR) in SMMC-7721 and SMMC-7721DOX. **c** OCR based on a mitochondrial stress test. **d** Effects of various drugs on the OCR in a mitochondrial stress test. **e** ECAR of the culture media in a glycolysis stress test. **f** Effects of various drugs on the ECAR in a glycolysis stress test. Significance: **p < 0.01 vs. SMMC-7721

Intracellular ATP levels and energy metabolism mechanisms were examined in SMMC-7721 and SMMC-7721/DOX cells. Cellular ATP levels in SMMC-7721/DOX were significantly greater than those in SMMC-7721 (Fig. 2a). In a mitochondrial stress test, the basal OCR in SMMC-7721/DOX cells was greater than that in SMMC-7721 cells. In response to oligomycin (Oli), an inhibitor of ATP synthase, the OCR was reduced in both cell types, and this reduction was greater for the resistant cells, indicating that SMMC-7721/DOX cells had higher ATP synthase activity and thereby had increased mitochondrial ATP production. In response to carbonyl carbonylcyanide-4-(trifluoromethoxy) phenylhydrazone (FCCP), the OCR increased for both cell types, and the maximal respiration in the resistant cells was much greater than that in SMMC-7721 cells, indicating that SMMC-7721/DOX had a strong spare respiratory capacity in OXPHOS (Fig. 2c and d). In a glycolysis stress test, the ECAR was increased in the culture media of the two kinds of cells, providing evidence for glycolysis. In response to Oli, the increase in ECAR was significantly greater for SMMC-7721/DOX than SMMC-7721 cells, suggesting that SMMC-7721 cells had a greater glycolytic capacity when OXPHOS was inhibited. In response to 2-deoxy-d-glucose (2-DG), as expected, glycolysis was significantly inhibited in the two cell types (Fig. 2e and f). These results suggested that glycolysis was dominant in SMMC-7721 cells, and mitochondrial energy metabolism and glycolysis were both significantly increased in SMMC-7721/DOX cells (Fig. 2b). Glycolysis was dominant in SMMC-7721 cells, and mitochondrial energy metabolism and glycolysis were both significantly increased in SMMC-7721/DOX cells (Fig. 2b).

### Effects of blocking cell energy metabolism on cell viability and intracellular ATP levels

**Fig. 3:**
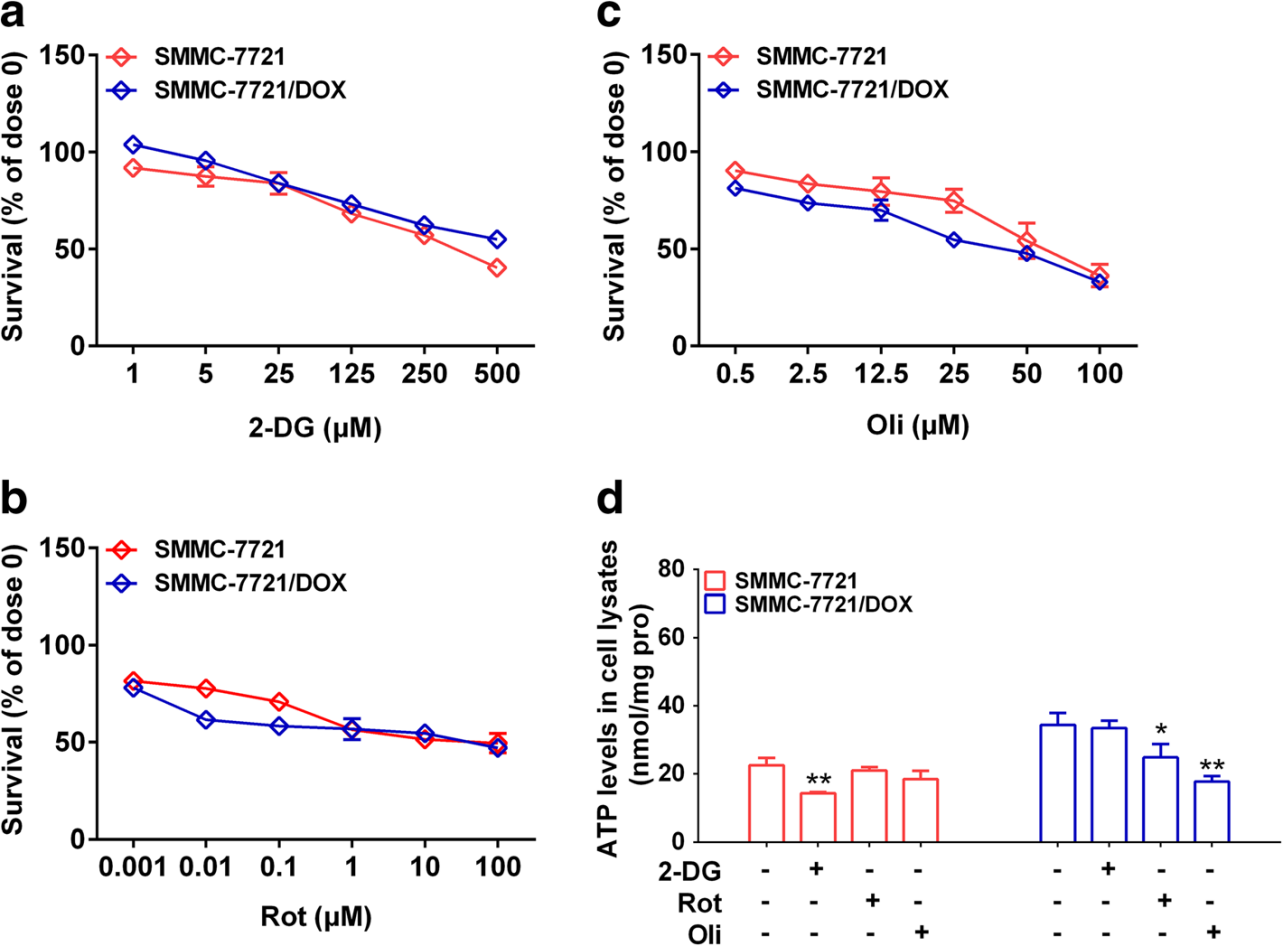
Effects of blocking cell energy metabolism on cell viability. **a** Effects of the inhibitor 2-DG on cell viability. **b** Effects of the inhibitor Rot on cell viability. **c** Effects of the inhibitor Oli on cell viability. **d** Effects of 2-DG, Rot, and Oli on intracellular ATP levels in SMMC-7721 and SMMC-7721/DOX cells. Significance: *p < 0.05, **p < 0.01 vs. Control

To explore the effects of glycolysis and OXPHOS on drug resistance, cell viability was assessed after treatment with 2-DG, rotenone (Rot, an inhibitor of respiratory chain complex I), and Oli at various concentrations for 24 h. 2-DG inhibited cellular activity of both cell types, and the IC_50_ of 2-DG was lower for SMMC-7721 than SMMC-7721/DOX, suggesting that SMMC-7721 cells depend on glycolysis (Fig. 3a, Table 2). In contrast, drug-resistant cells were sensitive to Rot and Oli (Fig. 3b and c, Table 2). Intracellular ATP levels was measured after treatment with 2-DG (5 μM), Rot (0.1 μM), and Oli (0.5 μM) for 24 h. 2-DG reduced the intracellular ATP level in SMMC-7721 cells, and Rot and Oli reduced the intracellular ATP levels in SMMC-7721/DOX cells (Fig. 3d). These findings indicated that the activation of OXPHOS is an essential characteristic of drug-resistant cells.

**Table 2:** IC_50_ of glycolysis inhibitors and OXPHOS inhibitors in hepatoma carcinoma cells

IC_50_ (μM)	SMMC-7721	SMMC-7721/DOX
2-DG	358.5	643.6
Rot	28.47	22.55
Oli	63.53	36.16

### Differences in α-KG content and the expression of α-KG-metabolizing enzymes between SMMC-7721 and SMMC-7721DOX cells

**Fig. 4:**
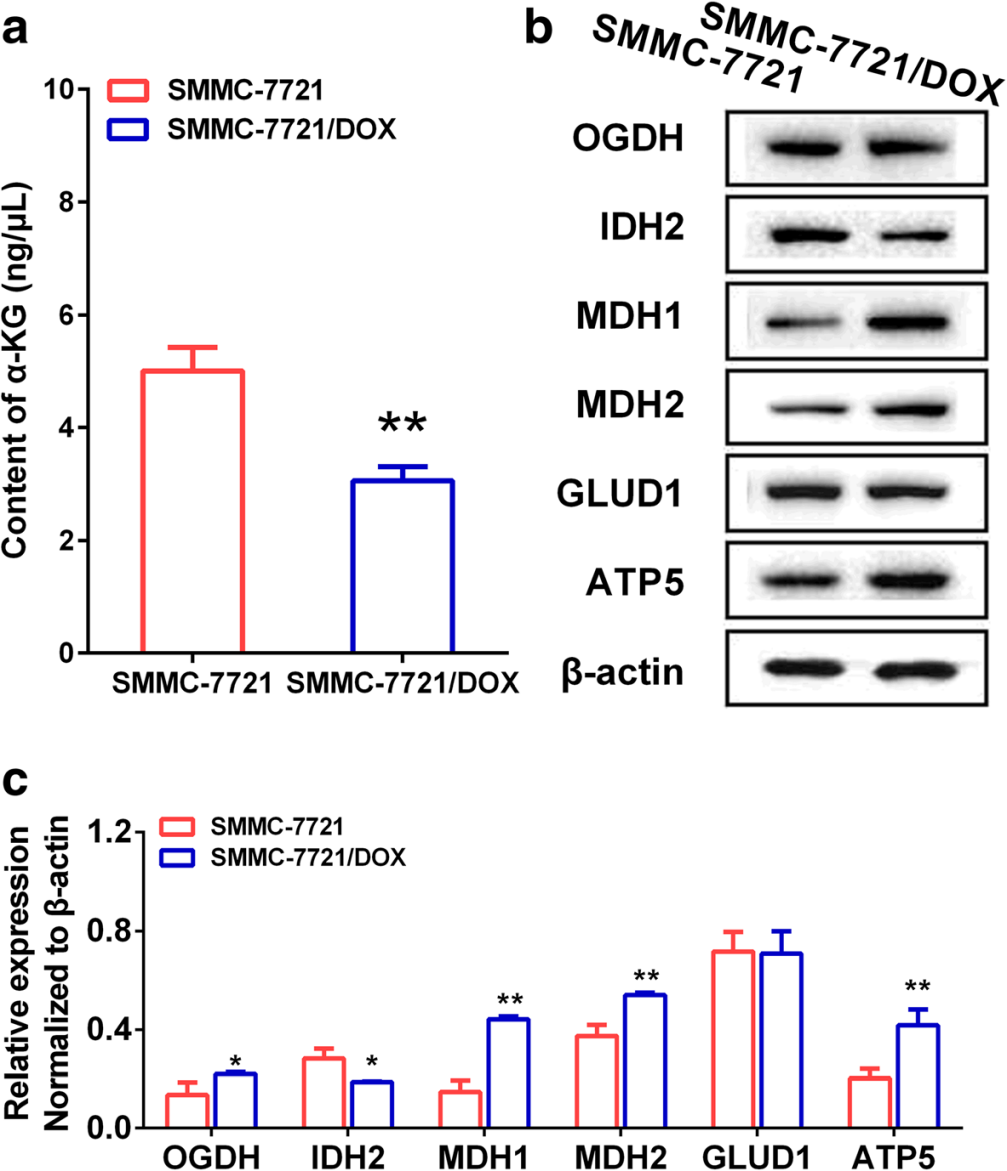
Differences in α-KG content and expression of α-KG-metabolizing enzymes between SMMC-7721 and SMMC-7721DOX. **a** α-KG content in SMMC-7721 and SMMC-7721/DOX cells. **b** Blots of OGDH, IDH, MDH1, MDH2, GLUD1, and ATP5 in SMMC-7721 and SMMC-7721/DOX cells. **c** Expression of OGDH, IDH, MDH1, MDH2, GLUD1, and ATP5 in SMMC-7721 and SMMC-7721/DOX cells. Significance: *p < 0.05, **p < 0.01 vs. Control

α-KG is an intermediate in the TCA cycle, but it is also a biologically active substance involved in OXPHOS and glycolysis via the regulation of ATP synthase and hypoxia inducible factor-1 (HIF-1). We investigated the relationship between α-KG and the activation of oxidative phosphorylation in drug-resistant cells. The α-KG content was significantly higher in drug-resistant cells than in SMMC-7721 cells (Fig. 4a). We examined the expression of ketoglutarate dehydrogenase (OGDH), isocitrate dehydrogenase 2 (IDH2), malic dehydrogenase (MDH1, MDH2), glutamate dehydrogenase 1 (GLUD1), and ATP synthase (ATP5), which are involved in α-KG metabolism. Compared with expression levels in SMMC-7721 cells (Fig. 4b, c, d, and e), OGDH, MDH1, MDH2, and ATP5 activity levels were higher, GLUD1 levels were not different, and IDH2 levels were lower in the drug-resistant cells. Consequently, the inhibition and activation of the malate-aspartate shuttle might be an important factor underlying the phenotypic differences in energy metabolism between SMMC-7721 cells and drug-resistant cells.

### Influence of α-KG on cell viability and intracellular ATP levels

**Fig. 5:**
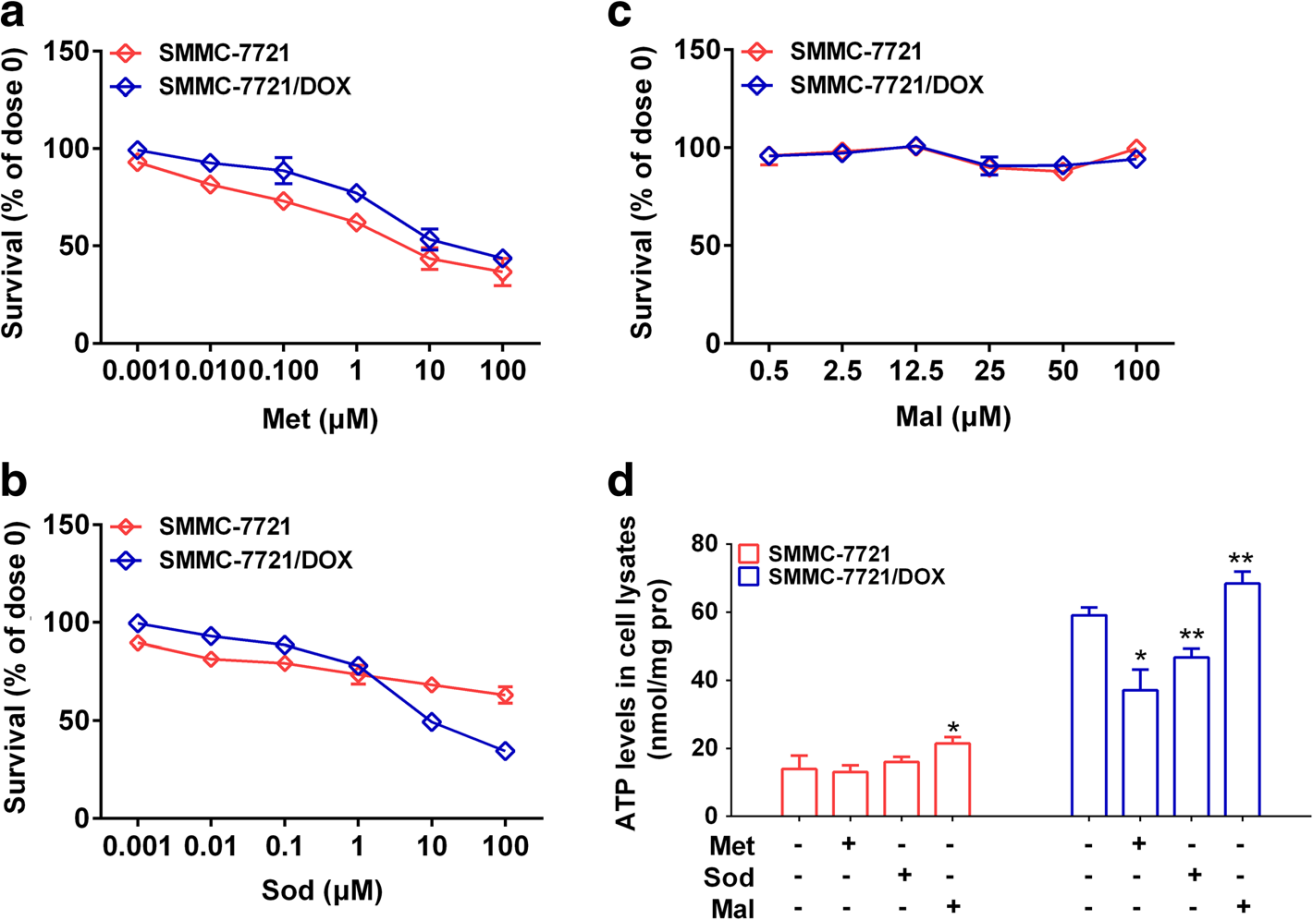
Effects of blocking cell energy metabolism on cell viability. **a** Effects of Met inhibition on cell viability. **b** Effects of Sod inhibition on cell viability. **c** Effects of Mal inhibition on cell viability. **d** Effects of Met, Sod, and Mal on intracellular ATP levels in SMMC-7721 and SMMC-7721/DOX cells. Significance: *p < 0.05, **p < 0.01 vs. Control

To explore the effect of α-KG on the drug resistance mechanism, cell viability was assessed after treatment for 24 h with methotrexate (Met), which inhibits OGDH and influences α-KG levels in the mitochondria, sodium oxamate (Sod), an inhibitor of the malate-aspartate shuttle affecting the α-KG distribution in the mitochondria via NADH metabolism, and l-(−)-malic acid (Mal), which can change the α-KG distribution in the mitochondria by activating the malate-aspartate shuttle. Met inhibited cellular activity in both cell types, and the IC_50_ of Met was lower for SMMC-7721 than SMMC-7721/DOX, suggesting a stronger inhibitory effect on SMMC-7721 (Fig. 5a, Table 2). Sod had a stronger inhibitory effect on SMMC-7721/DOX than on SMMC-7721 when the concentration was greater than 1 μM (Fig. 5b, Table 3). Mal had no significant effect on viability in either cell type in the range of 0.001 to 10 mM (Fig. 5c, Table 3). In general, the suppression of α-KG metabolism and transfer in the mitochondria could reduce the viability of hepatocellular carcinoma cells, and had a stronger inhibitory effect on resistant cells than non- resistant cells. Intracellular ATP levels were measured after treatment with Met (0.1 μM), Sod (1 μM), and Mal (1 μM) for 24 h. Met and Sod, which increase the accumulation of α-KG in mitochondria, significantly reduced ATP levels in the resistant cells, but had no effect on SMMC-7721 cells. Mal increased ATP levels in both cell types (Fig. 5d). These findings indicated that the accumulation of α-KG in mitochondria by the inhibition of metabolism or reduction in transfer can greatly reduce intracellular ATP levels in the drug-resistant cells.

**Table 3:** IC_50_ of regulators of α-KG metabolism in hepatoma carcinoma cells

IC_50_ (μM)	SMMC-7721	SMMC-7721/DOX
Met	6.23	29.82
Sod	61,526	149.7
Mal	–	–

### Influence of met on the energy metabolism mode

**Fig. 6:**
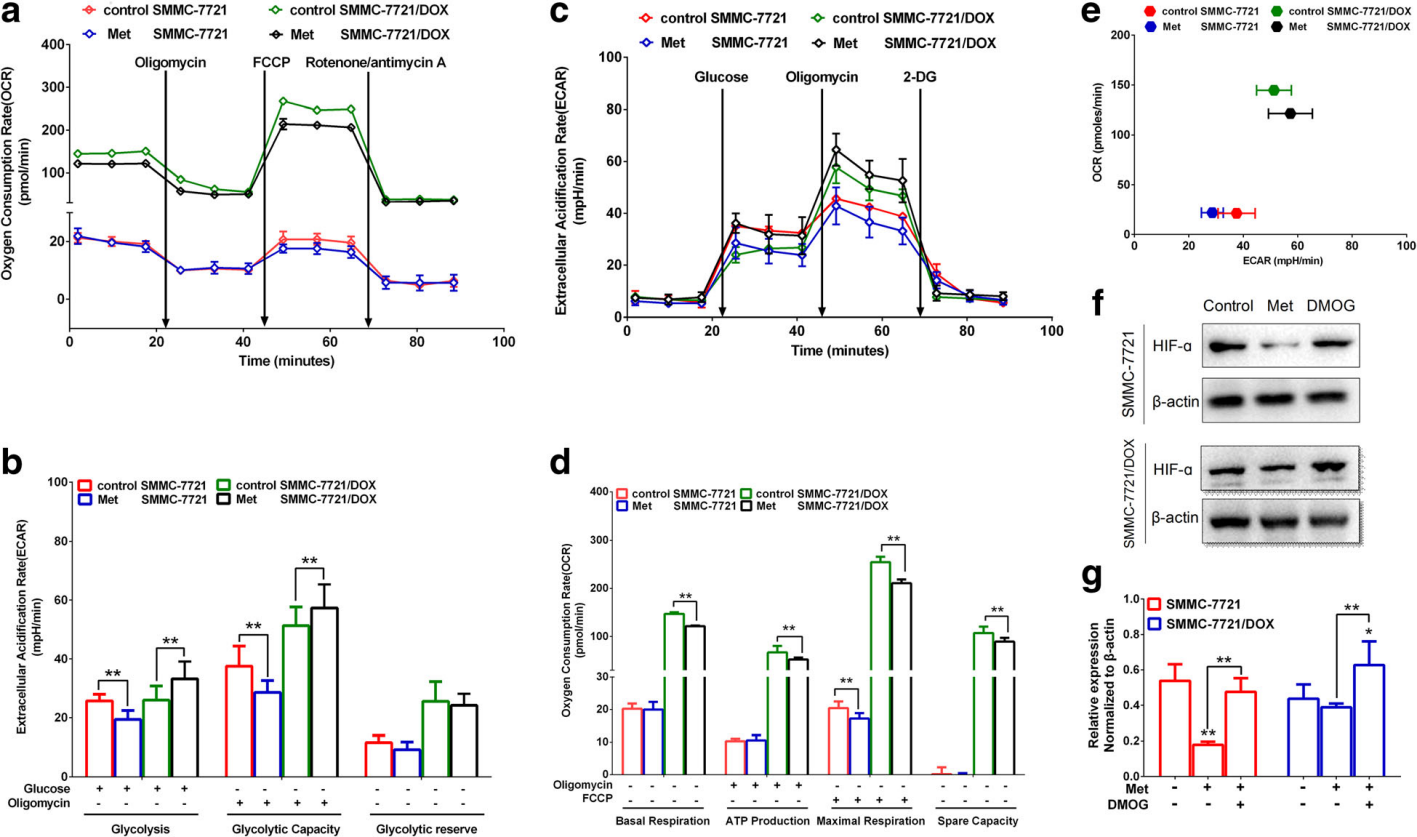
Influence of Met on the energy metabolism mode. **a** OCR based on a mitochondrial stress test. **b** Effects of Met on OCR in a mitochondrial stress test. **c** ECAR of the culture media in a glycolysis stress test. **d** Effects of Met on ECAR in a glycolysis stress test. **e** Cell respiration (OCR) against glycolysis (ECAR) in SMMC-7721 and SMMC-7721/DOX cells. **f, g** Expression of HIF-α in SMMC-7721 and SMMC-7721/DOX cells Significance: **p < 0.01 vs. SMMC-7721 or SMMC-7721/DOX

The basal oxygen consumption, ATP production, maximal respiration, and spare capacity in OXPHOS were significantly lower in SMMC-7721/DOX than in SMMC-7721, and only maximal respiration was reduced in SMMC-7721 cells when Met was used to inhibit OGDH activity and thereby reduce α-KG metabolism (Fig. 6a and b).

Glycolysis and the glycolytic capacity of SMMC-7721 were significantly decreased and glycolysis and glycolytic capacity of SMMC-7721/DOX were increased (Fig. 6c and d). It is possible that α-KG that entered the cytoplasm activated the proly-4-hydroxylase domain (PHD) and degraded hypoxia-inducible factor (HIF). In drug-resistant cells, the inhibition of α-KG metabolism can reduce the OXPHOS level and increase the glycolysis level, demonstrating that mitochondrial energy metabolism and glycolysis are coordinated, and the inhibition of mitochondrial energy metabolism can activate glycolysis (Fig. 6d).

This HIF-1α stabilization phenotype is similar to that after treatment of cells with desferrioxamine (DFO), an iron chelator, or dimethyloxalyglycine (DMOG), an established PHD inhibitor, but was not recapitulated with other α-KG analogues, such as Octyl-2KG, MPTOM001 and MPTOM002. Our study is the first example of an α-KG precursor which increases HIF-1αabundance and activity. We propose that DKG acts as a potent HIF-1α activator, highlighting the potential use of DKG to investigate the contribution of the PHD2-HIF-1α pathway to tumor biology.

### Influence of sod on the energy metabolism mode

**Fig. 7:**
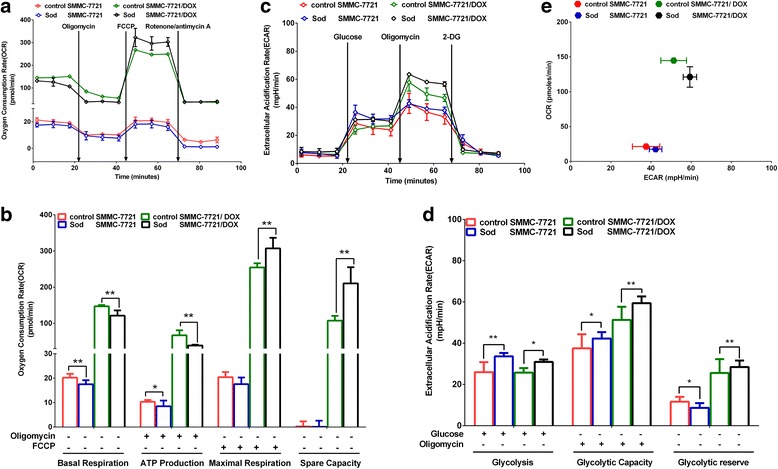
Influence of Sod on the energy metabolism mode. **a** OCR in a mitochondrial stress test. **b** Effects of Sod on OCR in a mitochondrial stress test. **c** ECAR of the culture media in a glycolysis stress test. **d** Effects of Sod on ECAR in a glycolysis stress test. **e** Cell respiration (OCR) against glycolysis (ECAR) in SMMC-7721 and SMMC-7721/DOX cells. Significance: *p < 0.05, **p < 0.01 vs. SMMC-7721 or SMMC-7721/DOX

When Sod was used to suppress the malate-aspartate shuttle to reduce the α-KG transfer, the basal oxygen consumption and ATP production of SMMC-7721 and SMMC-7721/DOX decreased significantly (Fig. 7a and b), and glycolysis and the glycolytic capacity increased significantly (Fig. 7c and d), which may be explained by reduced inhibitory effects on PHD and HIF. However, Sod significantly increased the maximal respiration, spare capacity in OXPHOS, and glycolytic reserve for SMMC-7721/DOX cells, indicating that the accumulation of α-KG had inhibitory effects on ATP synthase, and mitochondrial energy metabolism and glycolysis can be activated by suppressing the malate-aspartate shuttle (Fig. 7e).

### Influence of mal on the energy metabolism mode

**Fig. 8:**
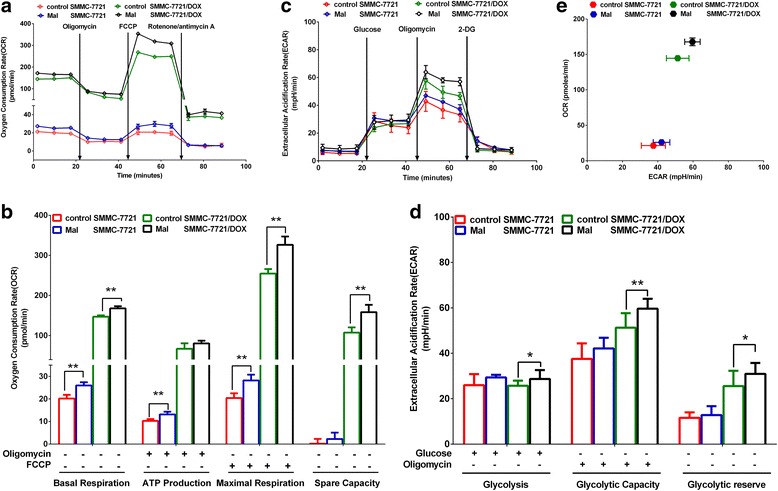
Influence of Mal on the energy metabolism mode. **a** OCR in a mitochondrial stress test. **b** Effects of Mal on OCR in a mitochondrial stress test. **c** ECAR of the culture media in a glycolysis stress test. **d** Effects of Mal on ECAR in a glycolysis stress test. **e** Cell respiration (OCR) against glycolysis (ECAR) in SMMC-7721 and SMMC-7721/DOX cells. Significance: *p < 0.05, **p < 0.01 vs. SMMC-7721 or SMMC-7721/DOX

Basal oxygen consumption, ATP production, maximal respiration, and spare capacity in OXPHOS increased in SMMC-7721 and SMMC-7721/DOX cells (Fig. 8a and b), and glycolysis, glycolytic capacity, and glycolytic reserve increased (Fig. 8c and d) when Mal was used to activate the malate-aspartate shuttle to increase the α-KG transfer. Mal could promote mitochondrial energy metabolism and glycolysis (Fig. 8e).

### Influence of met, sod, and mal on the α-KG content and expression of key enzymes involved in α-KG metabolism and transfer

**Fig. 9:**
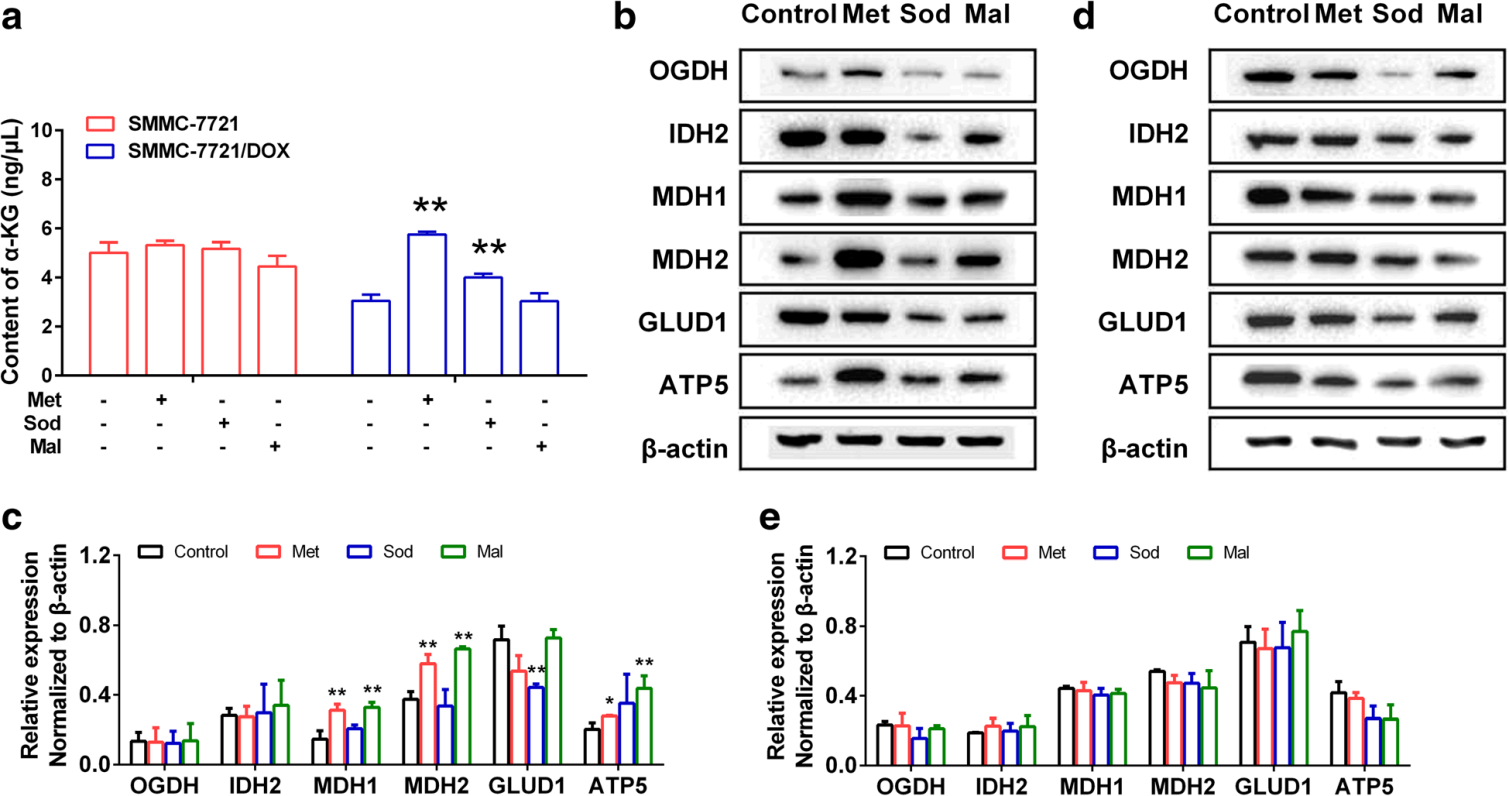
Influence of Met, Sod, and Mal on the α-KG content and expression of key enzymes involved in α-KG metabolism and transfer. **a** α-KG content in SMMC-7721 and SMMC-7721/DOX. **b**, **c** Blots of OGDH, IDH, MDH1, MDH2, GLUD1, and ATP5 in SMMC-7721. **d**, **e**. Expression of OGDH, IDH, MDH1, MDH2, GLUD1, and ATP5 in SMMC-7721/DOX. Significance: *p < 0.05, **p < 0.01 vs. Control

The balance and transformation between mitochondrial energy metabolism and glycolysis could be affected by α-KG metabolism and transfer, but the role of key enzymes involved in α-KG metabolism and transfer is unclear. We first examined the α-KG content and found that Met and Sod increased the α-KG content in SMMC-7721/DOX cells (Fig. 9a). We further examined the expression of key enzymes, including OGDH, IDH2, MDH1, MDH2, GLUD1, and ATP5. Met increased the expression of MDH1, MDH2, and ATP5 in SMMC-7721 cells (Fig. 9b and c), and had no significant influence on the expression of these enzymes in SMMC-7721/DOX cells (Fig. 9d and e). Sod significantly reduced the expression of GLUD1 in SMMC-7721 cells (Fig. 9b and c). Mal increased the expression of MDH1, MDH2, and ATP5 in SMMC-7721 cells (Fig. 9b and c), but had little influence on the expression of these enzymes in SMMC-7721/DOX cells (Fig. 9d and e). These results suggested that the inhibition of α-KG metabolism or regulation of α-KG transfer in SMMC-7721 cells could not only regulate mitochondrial energy metabolism and glycolysis, but could also trigger feedback in metabolic enzyme expression. However, in SMMC-7721/DOX cells, α-KG-metabolizing enzyme expression was not affected.

### Influence of met, sod, and mal on the content of DOX and resistance index

**Fig. 10:**
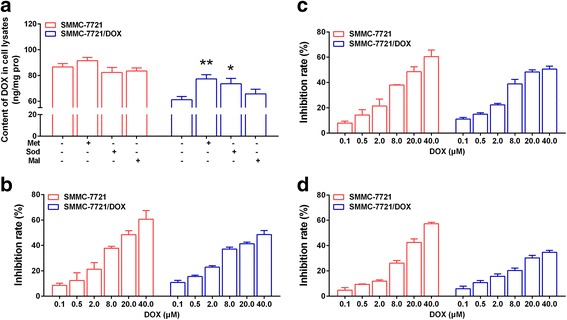
Influence of Met, Sod, and Mal on the DOX content and resistance index. **a** Effects of Met, Sod, and Mal on the DOX content in SMMC-7721 and SMMC-7721/DOX cells. **b** Effects of Met on the resistance index. **c** Effects of Sod on the resistance index. **d** Effects of Mal on the resistance index. Significance: *p < 0.05, **p < 0.01 vs. Control

We developed a novel and simple UPLC-MS/MS method to evaluate DOX (Supplementary Material). The results indicated that there was no significant effect of Met, Sod, and Mal on the DOX content in SMMC-7721 cells. However, in SMMC-7721/DOX cells, the inhibition of α-KG metabolism or the malate-aspartate shuttle could significantly increase intracellular DOX, which increases the sensitivity to DOX and reduces the resistance index (Fig. 10a-c, Table 4). The activation of the malate-aspartate shuttle had no significant effect on intracellular DOX levels, reduced the sensitivity to DOX, and improved the resistance index (Fig. 10a and d, Table 4).

**Table 4:** IC_50_ and RI of DOX after treatment with Met, Sod, and Mal

IC_50_ (μM)	SMMC-7721	SMMC-7721/DOX	RI
DOX	23.27	185.00	7.95
Methotrexate	20.37	46.81	2.30
Sodium oxamate	20.33	29.87	1.47
l-(−)-Malic acid	29.55	240.90	8.15

## Discussion

We examined the mechanisms of drug resistance from the perspective of energy metabolism in liver cancer cells, with a focus on the role of α-KG. It is well known that energy metabolism reprogramming is an important characteristic of tumor cells and exhibits substantial heterogeneity and variability. According to the results of this study, mitochondrial energy metabolism and the spare capacity in OXPHOS were weak in SMMC-7721 cells. When OXPHOS was inhibited, viability and intracellular ATP levels were not affected in SMMC-7721 cells. In contrast, when glycolysis was inhibited, viability and intracellular ATP levels were significantly reduced in SMMC-7721 cells. When liver cancer cells acquired drug resistance, mitochondrial energy metabolism and glycolysis increased significantly. In drug-resistant cells, viability and intracellular ATP levels could be significantly reduced by inhibiting OXPHOS.

α-KG is an important intermediate in the TCA cycle and participates in mitochondrial energy metabolism by regulating ATP synthase, PHD, and HIF. Owing to its role in the transition between energy metabolism modes, α-KG is involved in aging, tumorigenesis, and tumor drug resistance.

The regulatory effect of α-KG in mitochondrial energy metabolism has been attributed to its impact on ATP synthase, and the regulatory effect of α-KG on glycolysis has been ascribed to its impact on the activity of PHD and HIF in the cytoplasm. How do α-KG metabolism and distribution affect energy metabolism in drug-resistant cells? In SMMC-7721, the inhibition of α-KG metabolism in mitochondria enhanced the function of the malate-aspartate shuttle, which did not influence the level of OXPHOS, but significantly inhibited glycolysis. The inhibition of α-KG in the cytoplasm further inhibited OXPHOS and enhanced glycolysis. In Smmc-7721/DOX cells, the inhibition of α-KG metabolism or its distribution to the cytoplasm could reduce the function of OXPHOS and strengthen glycolysis. A decrease in intracellular ATP levels suggested a coordinated transformation of energy metabolism and the metabolic phenotype. Conversely, the activation of the malate-aspartate shuttle could increase intracellular ATP levels. In particular, OXPHOS and glycolysis levels were significantly higher in the drug-resistant cells than in non-resistant cells.

## Conclusions

**Fig. 11:**
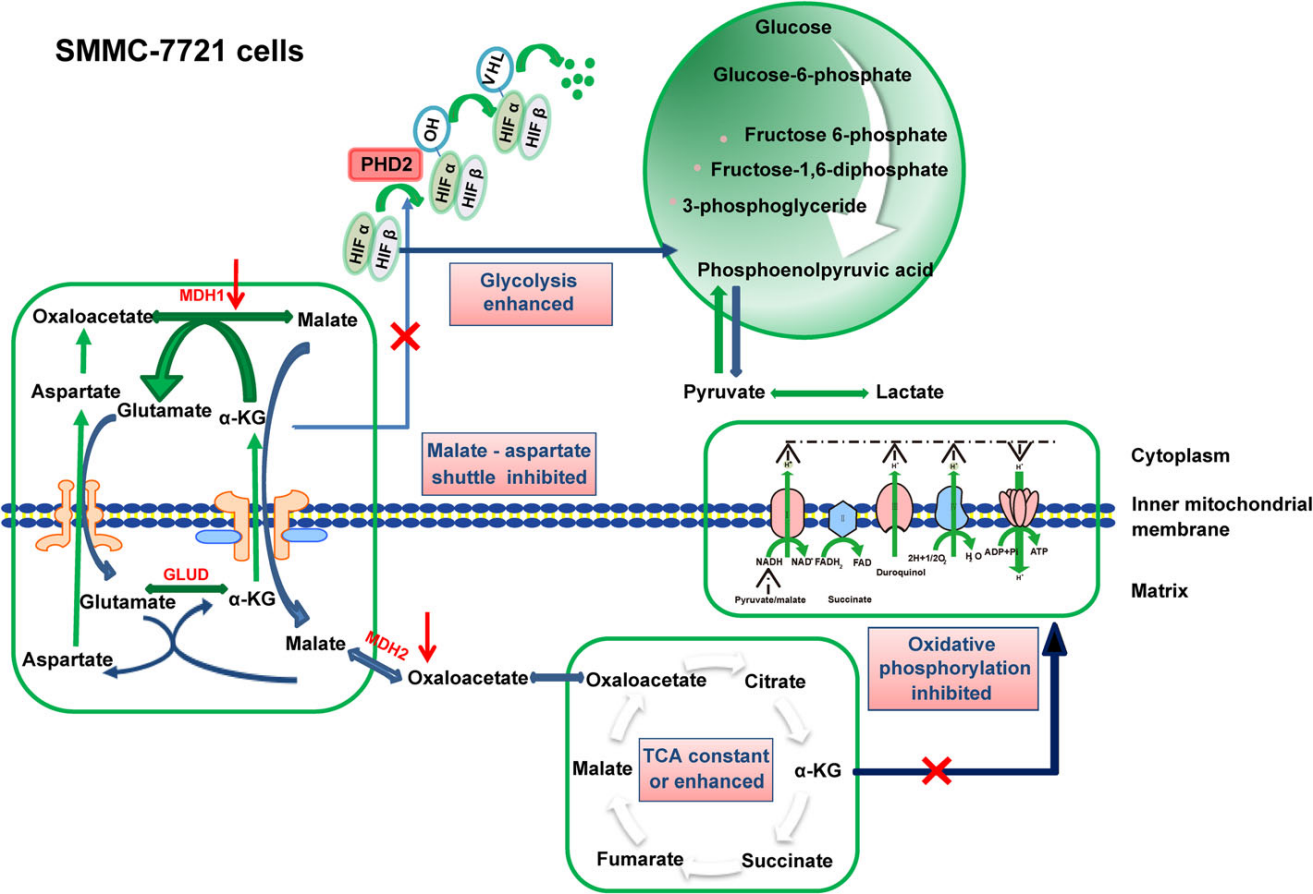
Schematic diagram of the energy metabolism mechanism in SMMC-7721 cells

**Fig. 12:**
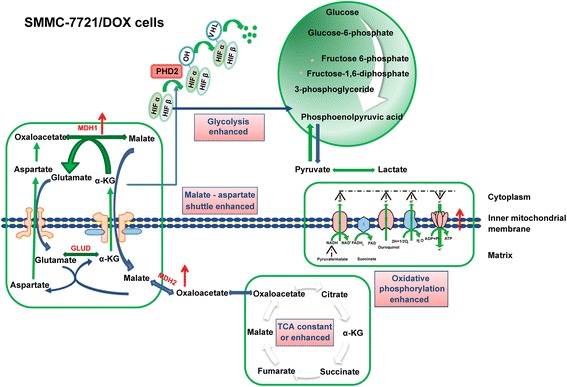
Schematic diagram of the energy metabolism mechanism in SMMC-7721/DOX cells

In conclusion, the results of this study suggested that the inhibitory effect of α-KG on ATP synthase was uncoupled with the TCA cycle and OXPHOS in SMMC-7721 cells, for which glycolysis was the main energy source (Fig. 11). In drug-resistant cells, a remarkable reduction of the inhibitory effects of α-KG on ATP synthase improved the coordination among TCA, OXPHOS, and glycolysis, and this may be associated with strong malate-aspartate shuttling, promoting the timely metabolism of intermediate products (Fig. 12).

## Abbreviations

2-DG: 2-deoxy-d-glucose
ATP: Adenosine triphosphate
ATP5: ATP synthase
DOX: Doxorubicin
ECAR: Extracellular acidification rate
FCCP: Carbonyl carbonylcyanide - 4 - (trifluoromethoxy) phenylhydrazone
GLUD1: Glutamate dehydrogenase 1
HIF-1: Hypoxia inducible factor-1
IDH2: Isocitrate dehydrogenase 2
Mal: l-(−)-malic acid
MDH: Malic dehydrogenase
MDR: Multidrug resistance
Met: methotrexate
MRM: Multiple reactions monitoring
OCR: Oxygen consumption rate
OGDH: Ketoglutarate dehydrogenase
Oli: Oligomycin
OXPHOS: Oxidative phosphorylation
PHD: Proly-4-hydroxylase domain
RI: Resistance index
Rot: Rotenone
Sod: Sodium oxamate
TCA: Tricarboxylic acid cycle
α-KG: α-Ketoglutaric acid
